# A Serological Survey of Paratuberculosis in the Polish European Bison (*Bison bonasus*) Population in 2018–2021

**DOI:** 10.3390/ani11072094

**Published:** 2021-07-14

**Authors:** Anna Didkowska, Marcin Ptak, Katarzyna Opałczyńska, Marlena Wojciechowska, Wanda Olech, Krzysztof Anusz

**Affiliations:** 1 Department of Food Hygiene and Public Health Protection, Institute of Veterinary Medicine, Warsaw University of Life Sciences (SGGW), Nowoursynowska 166, 02-787 Warsaw, Poland; marcin.ptak@protonmail.com (M.P.); katarzyna.opalczynska@gmail.com (K.O.); krzysztof_anusz@sggw.edu.pl (K.A.); 2 Department of Animal Genetics and Conservation, Institute of Animal Sciences, Warsaw University of Life Sciences (SGGW), Ciszewskiego 8, 02-786 Warsaw, Poland; marlena_wojciechowska@sggw.edu.pl (M.W.); wanda_olech@sggw.edu.pl (W.O.)

**Keywords:** European bison, *Mycobacterium avium* spp. *paratuberculosis*, paratuberculosis, serological survey, wildlife

## Abstract

**Simple Summary:**

The European bison (*Bison bonasus*) is an endangered species which faces a number of health threats. One potentially dangerous disease is paratuberculosis, which can cause diarrhea and cachexia in animals and is a potentially dangerous disease for humans. The aim of this study was to conduct a serological survey of paratuberculosis in Polish bison herds. Of the tested 165 European bison, three were found to be positive, indicating that paratuberculosis is not currently an urgent problem in this population. However, as the appearance of symptomatic paratuberculosis in a single European bison subpopulation could be fatal for the restitution of the species as whole, further random checks are needed.

**Abstract:**

The European bison (*Bison bonasus*) is an endangered species which faces a range of health threats. As little is known of exposure of European bison to paratuberculosis caused by *Mycobacterium avium* spp. *paratuberculosis*, known to cause losses in cattle, the aim of the present study was to conduct serological survey in Polish bison herds. Between September 2018 and February 2021, blood samples were collected from 165 European bison from different regions of Poland. Samples were taken whenever the animals were immobilized (e.g., putting on telemetry collars) as well as from any dead animal. The serum samples were tested with ELISA. Three individuals, originating from different captive herds, were found to be seropositive. In conclusion, it was found that *Mycobacterium avium* subsp. *paratuberculosis* infections are not currently a problem in European bison, especially in free-range herds.

## 1. Introduction

The European bison (*Bison bonasus*) is an endangered species which faces a wide range of population management problems [1,2] as well as environmental and health threats [3,4,5]. More than a quarter of the world’s population lives in Poland. Currently, the total number of European bison in Poland is 2316 (212 captive and 2104 free-living) (Bialowieski Park Narodowy—European Bison Pedigree Book issues edited after (bpn.com.pl, accessed on 1 June 2021)) and the population is gradually increasing. However, this increasing density favors the spread of infectious diseases, and hence there is a pressing need to implement active protection measures and carefully monitor the spread of any potential threats [6].

Among bacterial diseases, the most dangerous threat in recent years for European bison has been the outbreak of bovine tuberculosis (BTB) which, in this species, is mainly caused by *Mycobacterium caprae*. However, atypical mycobacteria have also been recently isolated from European bison lymph nodes [7]. The most common emerging atypical mycobacterium in cattle seems to be *Mycobacterium avium* spp. *paratuberculosis* (MAP) [8]. Not only does it cause serious economic losses in livestock production, but is also a potentially zoonotic agent [9]. MAP is an etiological agent of paratuberculosis (Johne’s disease) which has been noted in a number of types of livestock, as well as wild animal species [10,11,12]. Infections usually persist in a subclinical state for several years, and when clinical signs occur, they usually concern the digestive system (diarrhea) [12].

Arguably the most attractive way to monitor disease, especially in wildlife, is the serological survey; it is a relatively cheap and fast method which allows for the simultaneous examination of many individuals, and the performance of retrospective studies. It should be emphasized that the diagnosis of paratuberculosis may be more complicated in the case of infection with other mycobacteria, including those belonging to the *Mycobacterium tuberculosis* complex [13]. Additionally, it should be emphasized that, while ELISA testing seems to be suitable for monitoring disease in wild populations, this is not the case for individual animals, especially when both IgM and IgG antibodies are not checked and when samples are collected during one-time sampling. Such testing is associated with a risk of false positive results, if obtained in small numbers.

The aim of the study was to conduct a serological survey of paratuberculosis in the European bison (*Bison bonasus*) population in Poland and to figure out where there is a need for continuous monitoring of the disease.

## 2. Materials and Methods

### 2.1. Animal Sampling

The research was conducted as a part of the “Complex project of European bison conservation by State Forests” project in Poland; it is a comprehensive study of the cross-section of the Polish European bison population over several years. Samples were collected ante mortem whenever pharmacological immobilization of animals was performed for another purpose, such as when moving them to another bison center or putting on telemetry collars. All the procedures were carried out with high respect of animal welfare by qualified veterinarians in accordance with Polish law. Post mortem samples were collected during culls approved by the decision of Polish General Directorate for Environmental Protection or Regulation of Polish Ministry of the Environment, or from animals found dead.

**Figure 1:**
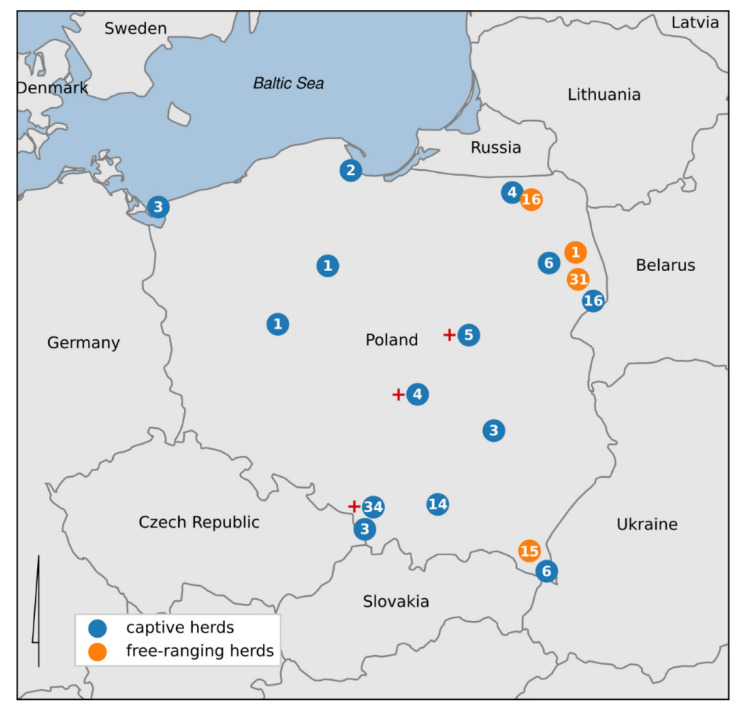
The map of Poland with marked captive and free-ranging European bison herds serologically tested for paratuberculosis (number of tested bison from each localization in a circle). The plus sign indicates populations where the individual with a positive test for *M. avium* spp. *paratuberculosis* antibodies were found.

Between September 2018 and February 2021, blood samples were collected from 165 European bison from free ranging (*n* = 63) and captive herds (*n* = 102) (Figure 1). Four out of the six free-living populations in the country were tested (Knyszyńska Forest (1/214), Borecka Forest (16/128), Bieszczady Mountains (15/707), and Białowieska Forest (31/715)). Of the captive herds, the following numbers of animals was tested in the largest centers: Pszczyna (34/56), Białowieża (16/27), Niepołomice (14/22), and Gdańsk Oliwa (2/13). Animals were tested in other captive herds, but in these, only fewer than 10 animals are kept. The sex division was as follows: 79 males and 85 females, with one individual not being identified (partly eaten carcass). The animals ranged in age from a few months to 23 years; however, 14 individuals were of unknown age (Table 1). 

Blood samples were collected ante mortem (after pharmacological immobilization) or from the jugular vein (*v. jugularis externa*), tail vein (*v. caudalis mediana*), or, in case of post mortem collection, from the heart. Blood was collected with a 1.2 mm-diameter needle into serum tubes with clot activator and centrifuged. The obtained serum samples were stored in the freezer at −20 °C.

### 2.2. Diagnostic Analaysis

After defrosting, the serum samples were tested with a *Mycobacterium paratuberculosis* Test Kit for Cattle PARACHEK^®^ 2 (Prionics AG, Schlieren-Zurich, Schlieren, Switzerland). The test is designed and standardized for cattle. However, ELISA tests for cattle are also commonly used in European bison [14,15] due to the close phylogenetic connection between those two species and the lack of tests designed specifically for European bison. The procedure was carried out in accordance with the manufacturer’s instructions. The absorbance of each well was read at a wavelength of 450 nm with an EPOCH spectrophotometer (BioTek Instruments Inc., Winooski, VT, USA). For interpreting the results, the same cut-off values were used as those given by the manufacturer of the test for cattle.

## 3. Results

### Paratuberculosis

From tested serum samples (*n* = 165), only three (1.81%) were found to be positive in the test for *M. avium* spp. *paratuberculosis* antibodies. All seropositive animals originated from captive herds: a one-year-old male from Pszczyna herd (56 herd members), a three-year-old male from Warsaw Zoo (seven members), and a 10-year-old female from Smardzewice Breeding Center (six members). Although other ruminants are kept in Pszczyna Herd and Warsaw Zoo, they do not have direct contact with the European bison. Interestingly, the seropositive bison from the Smardzewice Breeding Center had been confirmed to be *Mycobacterium caprae*-positive in other studies [7].

## 4. Discussion

Our findings indicate that antibodies against *M. avium* ssp. *paratuberculosis* are rarely present in European bison, indicating that they do not have much contact with this pathogen. Three seropositive animals were detected, but unfortunately their feces were not available to confirm or exclude infection by PCR. 

The present study is the first cross-sectional study of paratuberculosis in European bison population. The collected material is unique, particularly considering that the European bison is a wild and endangered species. Previous research was limited to 23 young individuals from one region of Poland, and no positive result was obtained [16]. As it might be expected, the three seropositive individuals identified in the present study came from captive herds, which is in line with previous scientific reports indicating that MAP infections are mainly associated with captive herds and zoo animals [17]. Although MAP infection is known to occur in farmed cervids, recent studies have examined the problem in zoos, in which the problem is believed to be underestimated. Infection has been noted in both ruminants and other species [18,19].

To the best of our knowledge, this is the first recorded detection of antibodies to MAP in European bison. However, cases of MAP infection have been noted in the closely related American bison (*Bison bison*) [20,21,22]. Furthermore, reports suggest that ruminants are the main reservoir of paratuberculosis [12], which places the European bison in the high-risk group for potential MAP infection. In the case of large ruminants, MAP affects the digestive system, with the most noticeable clinical sign in cattle being intense, watery diarrhea, not susceptible to treatment. It can be assumed that similar symptoms would be seen in European bison. While it is possible that vaccination may be used to manage an outbreak in a captive herd (see, e.g., [23]), the disease could lead to a rapid population reduction in the case of free-ranging herds. Some wild ruminant populations have demonstrated a high seropositivity, e.g., 17.64% in sika deer (*Cervus nippon*) in China [24], and 30.16% in Spanish red deer (*Cervus elaphus hispanicus*) [25], while much lower levels have been found in others, e.g., from 1.9% to 12.2% in free ranging cervids in Norway, depending on species [26].

In Poland, the largest group of ruminants are cattle. Although the prevalence of MAP-positive herds is estimated to be rather low in Poland, low numbers of cases are nevertheless regularly reported [27,28]. As disease transmission between species is relatively easy, e.g., through sharing common pastures, which is commonly known in Polish European bison herds [29], paratuberculosis should be viewed from a broader perspective, i.e., as a threat to all wildlife, including the European bison. Furthermore, such transmission should also take into account the economic aspects or potential public health implications [12].

The diagnosis of paratuberculosis is complicated, and ELISA is considered to be the most specific method to detect antibodies; however, higher sensitivity can be gained with fecal PCR, culture, and pathology. Nevertheless, both methods are equally effective and are used comparably frequently [12]. According to the manufacturer, the ELISA kit used in the present study has a test specificity of 99% for cattle, with a detection rate approaching 80% while the subject is still asymptomatic; however, the manufacturers also state that the history of the cattle herd can affect the calculations. While it would be reasonable to assume that the test would demonstrate similar specificity and sensitivity in European bison as in cattle, some studies have found ELISA tests to perform significantly less well in animals other than the target species [30].

Another limitation of this study is the small number of samples taken from most of the herds, which prevents any accurate of seroprevalence. However, given the endangered nature of European bison population, it is extremely difficult, or even impossible, to randomly select a given number of individuals from all herds in a country. Therefore, the best method for monitoring the contact of population with pathogens could seem to be collecting material whenever an opportunity presents itself. Such large-scale serological investigations are also convenient in other, unendangered wildlife species, as material can be collected not only during planned immobilization, but also during hunting. However, it should be noted that serological tests are not the best method for examining individual animals, especially valuable animals, such as those from the zoo.

It cannot be ruled out that, due to the potential immunological cross-reactivity, for example, following contact with environmental mycobacteria, our results may have been false. Furthermore, such isolated single positive results should be interpreted with great caution in serological surveys. Interestingly, in one out of the three seropositive European bison, the MTBC complex was [7]. It cannot be excluded that this result was a false positive, and occurred as a result of MTBC infection. While little data exist on the impact of MTBC infection on the result of paratuberculosis testing, it is commonly known that *M. avium subsp. paratuberculosis* can influence the diagnosis of tuberculosis [31].

## 5. Conclusions

Our findings indicate that *Mycobacterium avium* subsp. *paratuberculosis* infections does not present a major problem, especially in free-range herds. It seems that there is currently no need for continuous monitoring of this disease in the Polish European bison population.

## Figures and Tables

**Table 1:** The numerical ratios of the studied European bison in terms of age, sex, and the type of herd.

Population	Age		Sex	
		Female	Male	Unknown
Captive herds	≤1 year	13	14	-
	2–5 years	22	23	-
	≥6 years	10	10	-
	Unknown	6	4	-
	Total	51	51	-
Free-range herds	≤1 year	-	2	-
	2–5 years	9	5	-
	≥6 years	23	20	-
	Unknown	2	1	1
	Total	34	28	1

## Data Availability

Raw data are available upon request from the corresponding author.
